# Supplementary material for: Network Pharmacology Analysis of the Potential Pharmacological Mechanism of a Sleep Cocktail
Source: Biomolecules. 2024 May 27;14(6):630. doi: 10.3390/biom14060630 (PMC11201840; doi:10.3390/biom14060630)
Supplement: Supplementary file 1 [file biomolecules-14-00630-s001.zip › Supplementary Figures.pdf]

Supplementary Figures

Network Pharmacology Analysis of the Potential Pharmacological Mechanism of a Sleep Cocktail

Yuyun Liang, Yanrong Lv, Jing Qin * and Wenbin Deng *
School of Pharmaceutical Sciences (Shenzhen), Sun Yat-sen University, Shenzhen 518107, China; liangyy56@mail3.sysu.edu.cn (Y.L.); lvyr8@mail.sysu.edu.cn (Y.L.)
* Correspondence: qinj29@mail.sysu.edu.cn (J.Q.); dengwb5@mail.sysu.edu.cn (W.D.)

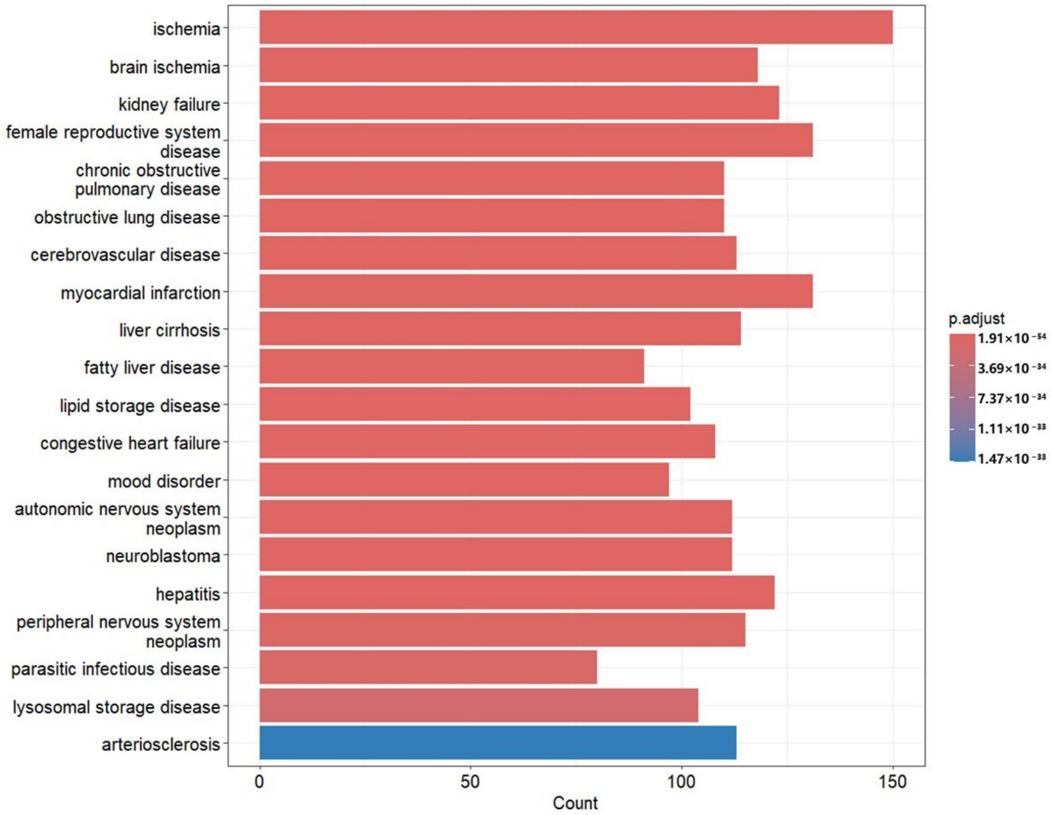

Figure S1. Disease oncology (DO) enrichment analysis of potential targets of sleep cocktail.

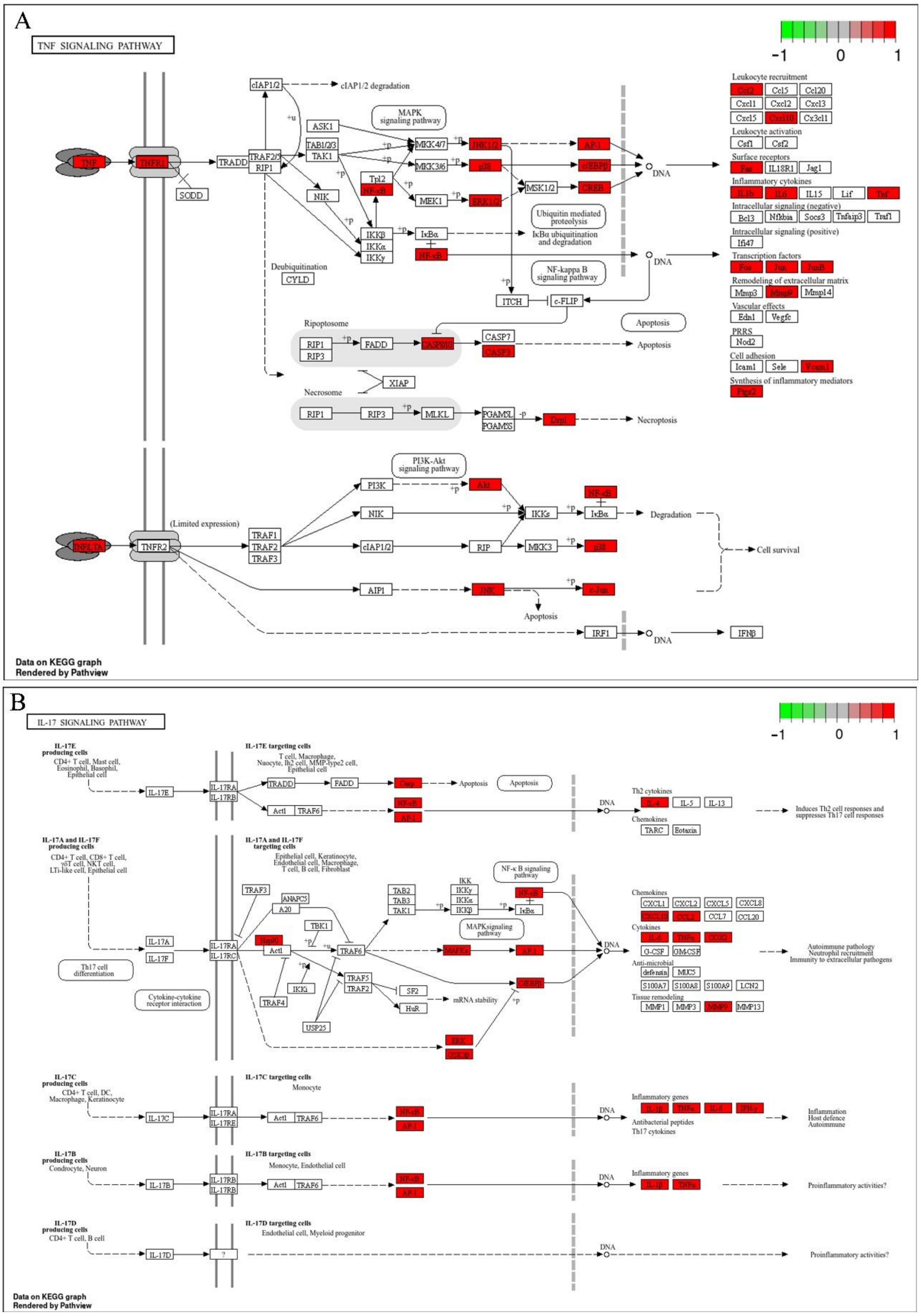

Figure S2. Graph of (A) TNF signaling pathway and (B) IL-17 signaling pathway. Red boxes denote common target genes.

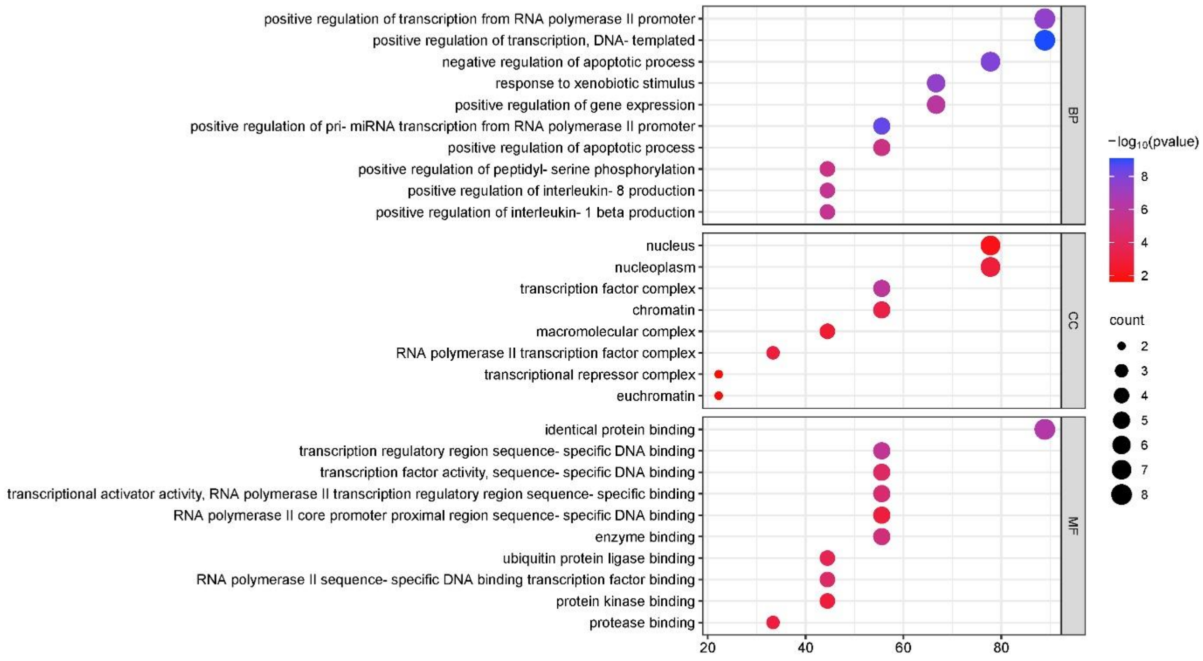

Figure S3. GO function analysis of nine hub genes.

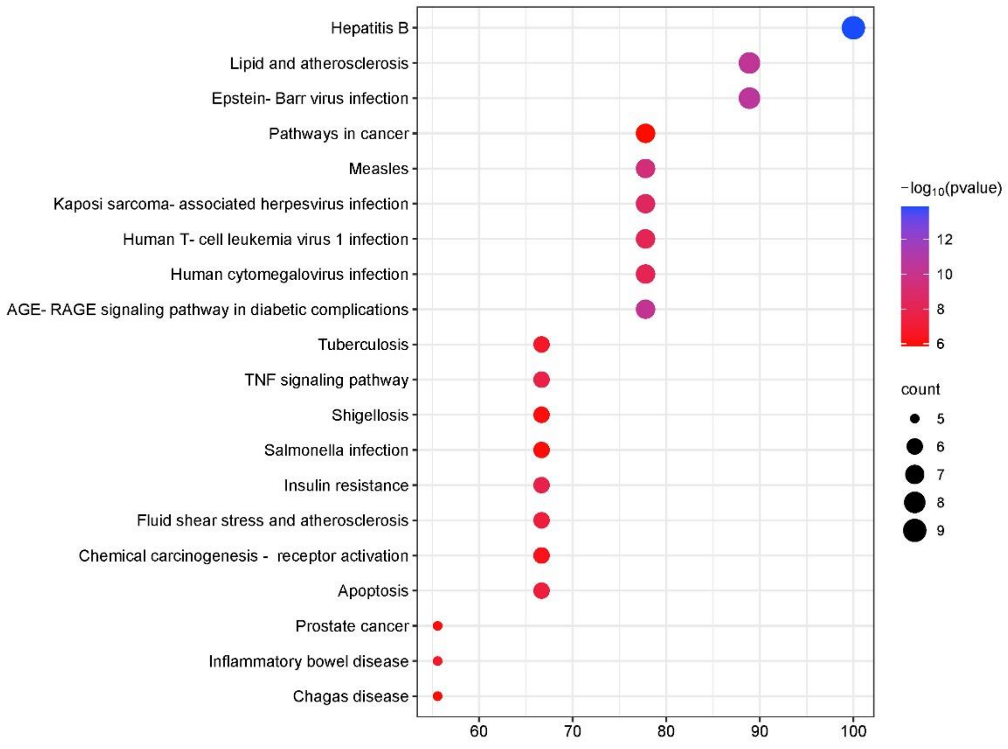

Figure S4. KEGG pathways enrichment analysis of nine hub genes.

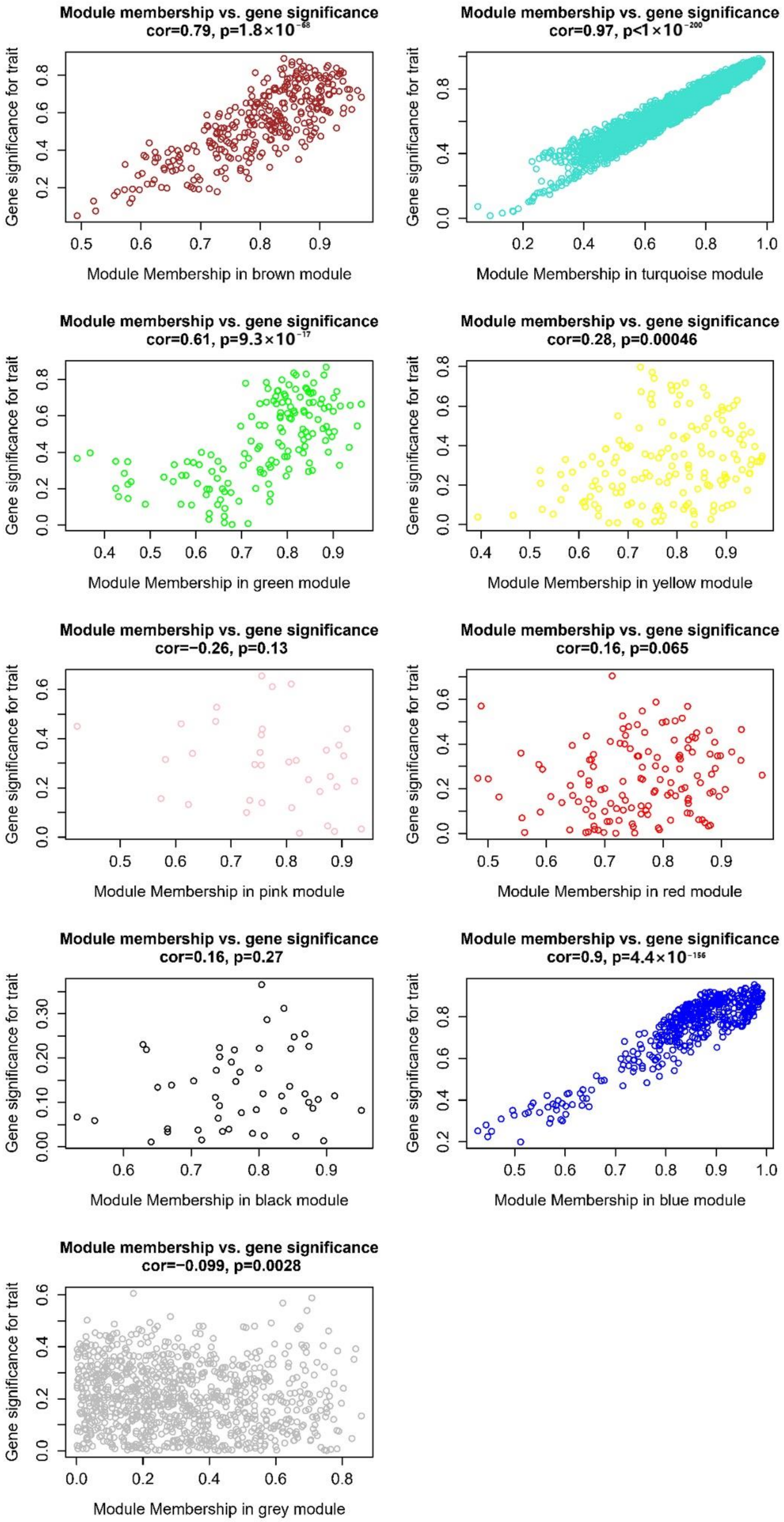

Figure S5. Scatterplot of MM and GS from all modules.

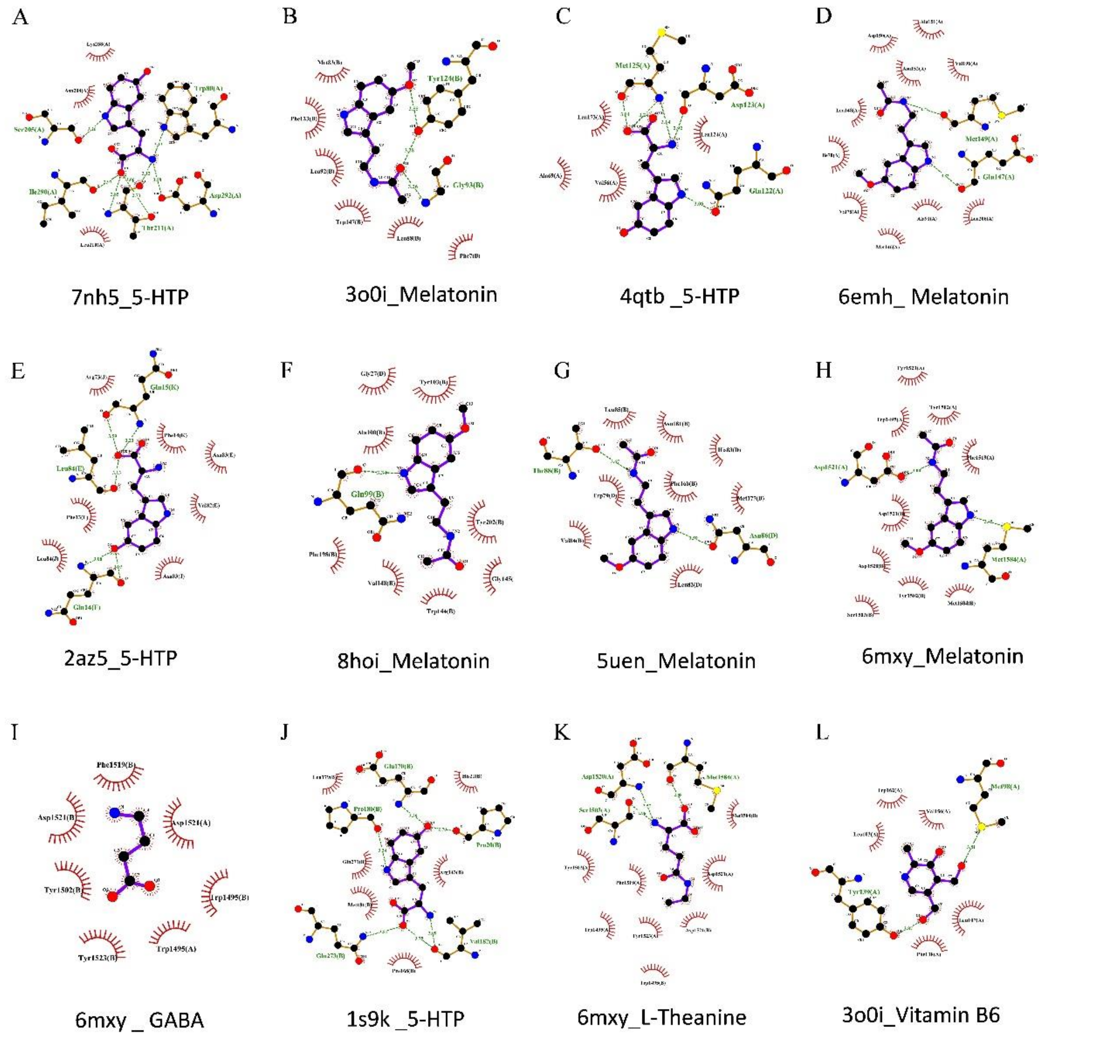

Figure S6. Molecular docking 2D diagram of key targets and active compounds: (**A**) 7nh5(AKT1)_5-HTP; (**B**) 3o0i(HSP90AA1)_Melatonin; (**C**) 4qtb(MAPK3)_5-HTP; (**D**) 6mxy(TP53)_Melatonin; (**E**) 2az5(TNF)_5-HTP; (**F**) 8hoi(BCL2)_Melatonin; (**G**) 5uen(ADORA1)_Melatonin; (**H**) 6emh_Melatonin; (**I**) 6mxy(TP53)_GABA; (**J**) 1s9k_5-HTP; (**K**) 6mxy(TP53)_L-Theanine; (**L**) 3o0i(HSP90AA1)_vitamin B6.
